# Effects of Crocin on The Pituitary-Gonadal Axis and Hypothalamic *Kiss-1* Gene Expression in Female Wistar Rats

**DOI:** 10.22074/ijfs.2018.5139

**Published:** 2018-01-15

**Authors:** Dina Zohrabi, Kazem Parivar, Mohammad Hossein Sanati, Nasim Hayati Roodbari

**Affiliations:** 1 Department of Biology, Science and Research Branch, Islamic Azad University, Tehran, Iran; 2 National Institute of Genetic Engineering and Biotechnology, Tehran, Iran

**Keywords:** Crocin, Folliculogenesis, Gonadal Steroid Hormones, Gonadotropins, *Kiss-1*

## Abstract

**Background:**

Saffron (*Crocus sativus L.*) has been traditionally used as a spice for coloring and flavoring in some countries cuisine. One of the main components of saffron is Crocin. Recent research have shown that crocin has various pharmacological effects. The aim of this study was to assess the effects of crocin on the Pituitary-Gonadal axis and *Kiss-1* gene expression in hypothalamus and ovarian tissue organization in female Wistar rats.

**Materials and Methods:**

In this experimental study, 18 adult female Wistar rats were randomly divided into three groups. Control group received normal saline and experimental groups received two different doses of crocin (100 and 200 mg/kg) every two days for 30 days. After the treatment period, blood samples were obtained from the heart and centrifuged. Next, the serum levels of follicle-stimulating hormone (FSH) and luteinizing hormone (LH), estrogen and progesterone hormones were measured by ELISA assay. The ovarian tissues were removed and fixed for histological investigation. The hypothalamic *Kiss-1* gene expression was measured using real-time polymerase chain reaction (PCR). All data were analyzed using one-way ANOVA.

**Results:**

A significant reduction (P=0.038) in the number of atretic graafian follicles (0.5 ± 0.31) was observed in rats treated with 200 mg/kg crocin. In addition, estrogen concentration in experimental groups (35.04 ± 0.85 and 36.18 ± 0.69 in crocin 100 and 200 mg/kg groups, respectively) compared to control group (38.35 ± 0.64) and progesterone concentration in rats treated with crocin 200 mg/kg (2.06 ± 0.07) compared to control group (2.16 ± 0.04), significantly decreased. Interestingly, relative expressions of *Kiss-1* mRNA significantly decreased in experimental groups (0.00053 ± 0.00051 and 0.0011 ± 0.00066 in crocin 100 and 200 mg/kg groups, respectively) (P=0.000) compared to control group (1 ± 0).

**Conclusion:**

Crocin, at hypothalamic level, reduces Kiss-1 gene expression and it can prevent follicular atresia and reduce serum levels of estrogen and progesterone.

## Introduction

Hypothalamic-pituitary-gonadal axis (HPG axis) has an important role in hormonal regulation of reproductive system. Disruption of this axis can have unpleasant consequences on fertility (1). Kisspeptin, also known as metastin, is a hypothalamic peptide encoded by the *Kiss-1* gene, which was first discovered as a metastasis inhibitor in melanoma cell lines (2). Recent studies have shown that the *Kiss-1* gene is also a key regulator of female gonadotropic axis in mammals (3) and is required for follicular development and ovulation during reproduction (4).

In rodents’ central nervous system, Kisspeptin expressing neurons were found in the anteroventral periventricular nucleus (AVPV) and arcuate nucleus (ARC) of the hypothalamus (5). Kisspeptin neurons send projections to gonadotropin- releasing hormone (GnRH) cell bodies, regulate the secretion of GnRH (6) and thereby control the release of follicle-stimulating hormone (FSH) and luteinizing hormone (LH) (7). Based on its role in sex organ development and the HPG-axis, kisspeptin neurons dysfunction can lead to abnormal fetal development and infertility (8).

Medicinal plants like *Pongamia pinnata, Trachyspermum ammi* and *Semecarpus anacardium* have shown to be able to improve fertility and resolve hormonal imbalances (9) while *Trigonella foenum, Carum carvi, Achyranthes aspera,* and *Rivea hypocrateriformis* have contradictory effects (10). So far, four types of phytochemicals including carotenoids, flavonoids, terpenoids and curcumins have been reported to be responsible for phytochemical activities of herbal drugs (11). Crocin is a carotenoid that is found in saffron (*Crocus sativus L.*). Saffron is traditionally has been used as a coloring or flavoring agent, as well as a herbal medicine (12). Moreover, previous studies have introduced crocin as an antioxidant (13), anticancer (14) and tumoricidal (15), anti-inflammatory (16), antinociceptive, antidepressant (17), and antianxiety agent (18). Crocin has been used as an effective treatment against Alzheimer’s disease (19), atherosclerosis (20), hyperlipidemia and hypertension (21). Considering various pharmacological properties of crocin, this study aimed to evaluate the effects of this phytochemical on female reproductive functions in Wistar rats.

## Materials and Methods

All aspects of animal care complied with the ethical guidelines and technical requirements approved by the Institutional Animal Ethics Committee. In this study, after two-week adaptation period, 18 virgin adult female Wistar rats were maintained under standard laboratory conditions. Rats (160-180 g) were housed under controlled lighting (12 hours light and 12 hours dark) at 20 ± 2°C and had free access to food and water. Synchronization of estrus in rats was performed using estradiol valerate and progesterone. Estrous cycle was monitored by vaginal smears.

In this experimental study, 18 Rats were randomly divided into three groups as follows: control group received 2 ml normal saline, experimental group I received crocin 100 mg/kg body weight (BW) intraperitoneally (Pharmaceutical Research Center, BuAli Research Institute, Mashhad University of Medical Sciences, Mashhad, Iran) and experimental group II received crocin 200mg/kg BW intraperitoneally every two days for 30 days (22).

## Hormonal assay

After the treatment period, animals in each group were anesthetized by ketamine/xylazine (k,80-100 mg/kg, X, 10-12.5 mg/kg) and blood samples were collected from their hearts. Blood samples were centrifuged for 10 minutes at 8000 rpm and the serum were separated and stored at -20°C. ELISA technique was used for evaluation of FSH, LH (commercial kits purchased from Pishtaz Teb Co., Iran), estrogen and progesterone hormones (commercial kits purchased from DRG Co., Germany).

## Histological investigation

The left ovary of animals were removed and fixed in 10% formalin solution. The specimens were processed through routine paraffin embedding method. Subsequently, 6 µm serial paraffin sections were stained with haematoxylin and eosin (H&E). The total number of sections was counted and the middle section of the ovary was determined. The follicles were counted in 5 sections per ovary which included the middle section and 4 sections from either side of the center. Ovarian follicle counting was performed (23) and atretic graafian follicles were identified (24) and counted using a light microscope (Olympus IX71, Japan).

## Evaluation of *Kiss-1* gene expression by quantitative real-time polymerase chain reaction

Brains were removed immediately from the skull. The hypothalami were dissected and frozen at -80°C. These tissues were thoroughly homogenized. Total RNA was extracted using RNeasy Mini Kit (Qiagen, Germany) and RNA concentration was determined by Nano Drop ND- 1000. cDNA synthesis was performed using 2 µg of total RNA and QuantiTect Reverse Transcription Kit (Qiagen, Germany). The quality of synthesized cDNA was assessed by polymerase chain reaction (PCR) using *ß-actin* gene. PCR products were qualified by electrophoresis on 1% agarose gel. Primers were designed by Primer premier 5 (Premier Bio Soft International, Palo Alto, CA, USA) software for the reference gene, *Kiss-1*. The rat *ß-actin* gene was used as the reference gene for data normalization (Table 1).

**Table 1:** Primers used for real-time quantitative polymerase chain reaction


Primer	Primer sequencing (5ˊ-3ˊ)	Product length (bp)	Annealing temperature (°C)

*Kiss-1*	F:TGCTGCTTCTCCTCTGTG	106	59
	R:ACGAGTTCCTGGGGTCC		
*β-actin*	F:CCATCTATGAGGGTTACGC	105	60
	R:TGTAGCCACGCTCGGTC		


Real-time PCR was performed in a thermal cycler Rotor gene 6000 (Corbett, AUS). The PCR mixture for each reaction contained 5 µl SYBR premix Ex Taq II, 0.5 µl of each primer (5 pmol/µl) and 50 ng cDNA adjusted to a final volume of 10 µl using DEPC water. All reactions were carried out in triplicate. The real-time PCR protocol included 5 minutes at 95°C followed by 40 repetitive cycles for 10 seconds at 95°C, 30 seconds at 60°C and 61°C for *Kiss-1* and 30 seconds at 72°C. The expression level of *Kiss-1* mRNA was normalized against *ß-actin* expression level as a housekeeping gene. The relative expression of *Kiss-1* gene was assessed using the ..Ct method and results were demonstrated as 2-..Ct based on previous reports (25, 26).

### Statistical analysis

The results were statistically analyzed using SPSS 19 software. Mean ± SD was calculated for each parameter and differences among means were evaluated by ANOVA followed by the Tukey post-hoc test using the Excel computer-based program. P<0.05 were considered statistically significant.

## Results

**Fig.1:**
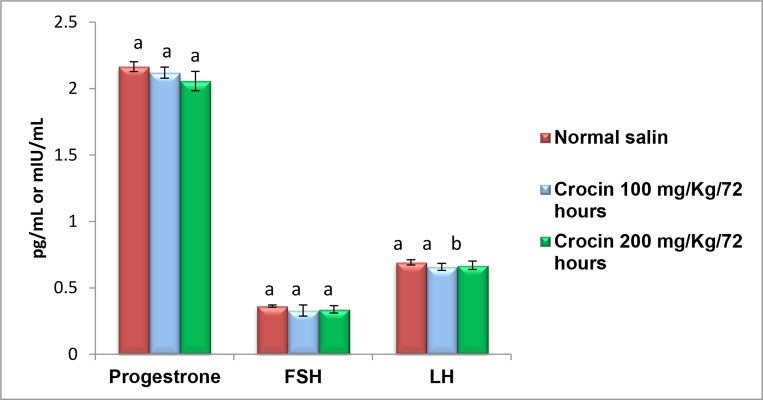
Comparison of serum FSH, LH, and progesterone levels among the experimental and control groups. Bars labeled with different letters are significantly different from each other at P<0.05. FSH; Follicle-stimulating hormone and LH; Luteinizing hormone.

**Fig.2:**
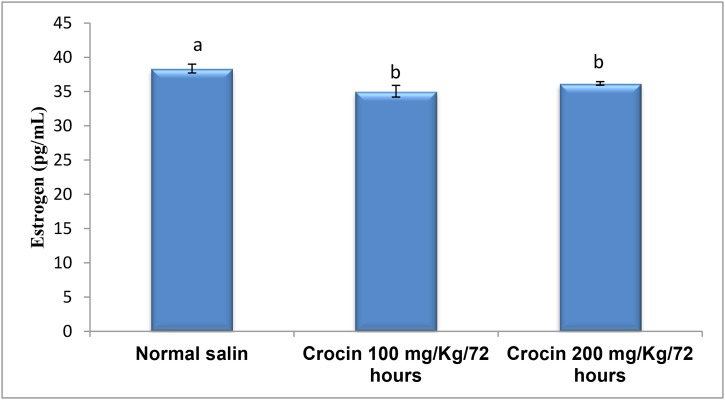
Comparison of serum estrogen level among the experimental and control groups. Significant differences were observed between groups treated with Crocin and control group (P<0.05).

**Fig.3:**
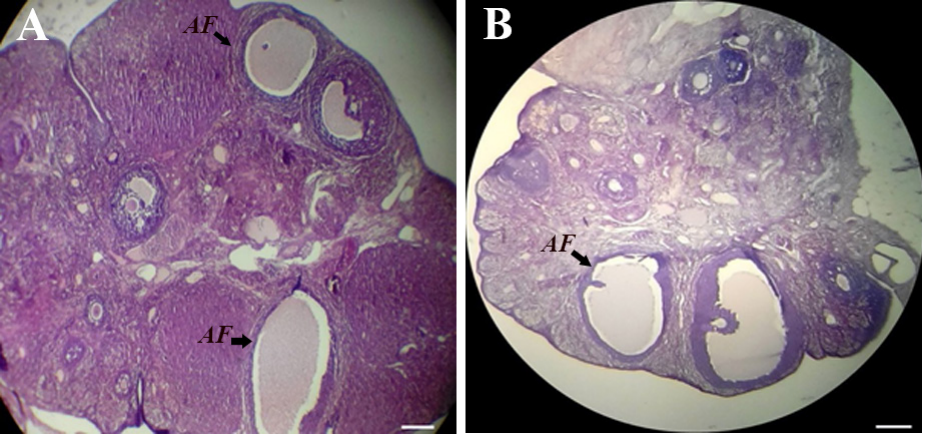
The ovarian tissue. Photomicrographs of ovary tissue (6-µm thick sections were stained with H&E; X100) in A. The control animals and B. Rats treated with 200 mg/kg crocin. AF; Atretic graafian follicle (scale bar: 100 µm).

There was no significant differences in FSH hormone levels between experimental groups (0.33 ± 0.042 for crocin 100 mg/kg, P=0.158 and 0.34 ± 0.073 for crocin 200 mg/ kg, P=0.302) and control group (0.36 ± 0.008). In addition, administration of crocin 100 and 200 mg/kg doses had no effect on LH levels in female rats ((0.66 ± 0.026, P=0.120) and (0.67 ± 0.032), P=0.350, respectivly) compared to control groups (0.69 ± 0.019) (Fig .1). However, both doses of crocin significantly decreased serum estrogen levels (35.04 ± 0.85 for crocin 100 mg/kg, P=0.000) and (36.18 ± 0.69 for crocin 200 mg/kg, P=0.000) compared to the control group (38.35 ± 0.64) (Fig .2). Also, progesterone concentrations significantly reduced in rats received 200 mg/kg crocin (2.06 ± 0.07, P=0.009) compared to control group (2.16 ± 0.04) (Fig .1). The expression of *Kiss-1* was significantly (P=0.000) reduced following treatment with crocin 100 and 200 mg/kg (Table 2). We also assessed the mean number of primordial, primary, growing, graafian and atretic graffian follicles and corpora lutea among rats treated with crocin 100 and 200 mg/kg compared to the control group. Apart from the number of atretic graafian follicles that was significantly lower in the rats treated with crocin 200 mg/kg (0.5 ± 0.31) compared to the control group (1.33 ± 0.45), we did not find any significant differences in the mean number of other follicles (Table 3). Figure 3 shows images of ovarian tissue in the control group and rats treated with crocin 200 mg/kg. 

**Table 2:** Mean hypothalamic *Kiss-1* gene expression in the experimental and control groups


Group	RF *Kiss-1* (mean ± SD)	P value compared to control group

Control	1 ± 0	-
Crocin (100 mg/kg)	0.00053 ± 0.0005^a^	0.000
Crocin (200 mg/kg)	0.0011 ± 0.0007^a^	0.000


All data were presented as mean ± SD. ^a^; Indicates a significant difference between experimental groups and control group (P<0.001).

**Table 3:** Mean number of primordial, primary, growing, atretic graafian follicles, graafian follicles and corpora lutea in the ovaries of rats in the experimental and control groups


Variable	Control	100 mg/kg/72 hours	200 mg/kg/72 hours	P value compared to control group

Primordial follicles	8.5 ± 1.169	5.8 ± 0.98	5.8 ± 1.17	0.0520.056
Primary follicles	2.17 ± 1.169	3.17 ± 0.408	1.33 ± 0.516	0.0970.183
Growing follicles	1.67 ± 0.516	1.17 ± 0.408	1.17 ± 0.408	0.1630.163
Graafian follicles	1.5 ± 0.837	1.17 ± 0.753	1.83 ± 0.753	0.7450.745
Atretic Graafian follicles	1.33 ± 0.448	1.33 ± 0.448	0.5 ± 0.31^a^	10.038
Corpora lutea	2.5 ± 0.548	4 ± 1.549	4 ± 1.265	0.1090.109


All data were presented as mean ± SD. ^a^; Indicates a significant difference between Crocin 200 mg/kg-treated group and control group (P<0.05).

Along with other hypothalamic factors, the *Kiss-1* gene regulates the release of GnRH and thereby controls the release of FSH and LH from the pituitary gland. FSH and LH control ovarian folliculogenesis .Therefore, an alteration in this axis may affect sex-related endocrine hormones and follicular development. In this study, we evaluated the effect of crocin on these parameters. The results revealed no significant differences in serum concentrations of FSH and LH, but the levels of estrogen following treatment with both doses of crocin and the level of progesterone following administration of crocin 200 mg/kg significantly reduced. 

Considering the fact that estrogen is secreted by follicular cells in the ovary, this may explain a role for crocin at the ovarian level. In this regard, previous studies have shown that carotenoids reduce the activity of cytochrome p450, thus inhibiting the transformation of cholesterol to pregnenolone, and consequently reducing the amount of estrogen. This effect of carotenoids is believed to be mediated by reduced expression of the *CYP19* gene which encodes an aromatase belonging to the cytochrome P450 family (27, 28). In addition, it has been shown that crocin reduces plasma levels of total cholesterol in a dose-dependent manner (29). Therefore, reducing estrogen and progesterone levels reported in the present study could be due to a reduction in cholesterol levels.

Considering the reduction in estrogen, an increase in *Kiss-1* gene expression is expected. It is well established that *Kiss-1* gene expression is negatively regulated by circulatory estrogen. In contrast to this expectation, the relative expression of *Kiss-1* gene was significantly down-regulated by crocin; the underlying reason(s) for this observation should be investigated in future studies. Despite reduction in *Kiss-1* gene, we observed no changes in FSH and LH levels. These observations suggest that reduced expression of *Kiss-1* gene is likely counterbalanced by other mechanisms controlling GnRH release and thereby pituitary FSH and LH secretion. Previous study have shown that some parameters such as NPY, GABA, Glutamate, etc. affect GnRH neurons and reproductive axis (30).

Also, differential action of estrogen on *Kiss-1* neurons in the AVPV and ARC of the hypothalamus, may explain our results. Estrogen increases *Kiss-1* gene expression in the AVPV while it reduces the expression of *Kiss-1* gene in the ARC. Hypothetically, based on this mechanism, one might expect crocin to reduce the expression of *Kiss-1* gene in ARC but not in AVPV nucleus. However, we believe that this is very unlikely and further research is required to clarify mechanisms via which crocin reduces *Kiss-1* gene expression. 

Following hormonal evaluation, we assessed histological sections for any alteration in folliculogenesis. The results revealed no significant changes in the mean value of the number of different follicles between the control and treated groups except for a reduction in the number of atretic graafian follicles which were significantly reduced in crocin 200 mg/kg-treated group. This observation may be related to the anti-apoptotic effect of carotenoids, like crocin. Carotenoids up-regulate the expression of *Cx43* (31), which is dominantly expressed in granulosa cells and maintains the integrity of the follicle thus reducing follicular apoptosis (32). In this regard, previous studies indicated that crocin can inhibit apoptosis via increasing *Bcl2/Bax* expression ratio (33, 34).

Another process involved in the generation of atretic follicles is excessive production of reactive oxygen species (ROS). Assimopoulou et al. (13) showed that crocin has a marked radical-scavenging activity. In this regard, Soeda et al. (35) reported that crocin inhibits oxidative stress- induced cell death via a glutathione (GSH)-dependent mechanism. Also, Hosseinzadeh et al. (36) showed that crocin decreases malondialdehyde (MDA) generation. The role of crocin as an anti-apoptotic agent is well established in different systems (37, 38).

As ovarian follicles synthesize estrogen, one may expect that a decrease in the number of atretic follicles may result in an increase in estrogen production, which is contrary to results showing a reduction in estrogen levels. This effect might be due to a reduction in aromatase activity induced by crocin. The reduction in estrogen might be possibly due to crocin effect on the ovary rather than on the hypothalamus, as crocin had no effects on FSH and LH levels.

Based on the literature, crocin can induce the expression of genes like *XBP, BiP, CHOP, F4/80, TNF-a, NOS-2, IFN-a* (39), *Mmp-9, Cox-1, Cox-2, Bcl-2,* and *Bax* (40). In our study, crocin reduced the expression of the Kiss- 1gene which might be a result of crocin interaction with factors involved in regulation of gene expression.

## Conclusion

Our results revealed that at the hypothalamic level, crocin reduces *Kiss-1* gene expression; however, reduced *Kiss-1* gene expression did not affect sex-related hormones, indicating that other mechanisms may have counterbalanced this reduction. At the ovarian level, crocin acts as an anti-apoptotic agent and reduces follicular atresia. Crocin may also indirectly reduce aromatization via regulating genes involved in this process. Overall, these data suggest that crocin may interfere with factors regulating gene expression and this hypothesis needs further investigations.
